# Recommendations for selection, treatment, and follow-up in peptide receptor radionuclide therapy (PRRT) for neuroendocrine tumors: a Delphi consensus from the Galician Multidisciplinary Group on Neuroendocrine and Endocrine Tumors (GGNET)

**DOI:** 10.1007/s12094-026-04226-7

**Published:** 2026-03-10

**Authors:** Nieves Martinez-Lago, José Manuel Cabezas Agricola, Urbano Anido Herranz, Zulema Nogareda Seoane, Antía Cousillas Castiñeiras, Rafael Varela Ponte, Pablo Fernández Catalina, Estephany Abou Jokh Casas, José María de Matías Leralta, Virginia Pubul Nuñez

**Affiliations:** 1 Medical Oncology Department, Santiago de Compostela University Clinical Hospital (CHUS) and Health Research, Institute of Santiago de Compostela (IDIS), Santiago de Compostela, Spain; 2 Endocrinology Department, Santiago de Compostela University Clinical Hospital (CHUS) and Health Research Institute of Santiago de Compostela (IDIS), Santiago de Compostela, Spain; 3 Department of Psychiatry, Radiology, Public Health, Nursing and Medicine, Universidade de Santiago de Compostela (USC), Santiago de Compostela, Spain; 4 Nuclear Medicine Department, Santiago de Compostela University Clinical Hospital (CHUS) and Health Research Institute of Santiago de Compostela (IDIS), Santiago de Compostela, Spain; 5 Medical Oncology Department, Complexo Hospitalario Universitario de Pontevedra, SERGAS, Pontevedra, Spain; 6 Radiology Department, Santiago de Compostela University Clinical Hospital (CHUS) and Health Research Institute of Santiago de Compostela (IDIS), Santiago de Compostela, Spain; 7 Endocrinology Department, Complexo Hospitalario Universitario de Pontevedra, SERGAS, Pontevedra, Spain; 8 Nuclear Medicine Department, Hospital Universitario Lucus Augusti, SERGAS, Lugo, Spain; 9 Endocrinology Department, Hospital Universitario Lucus Augusti, SERGAS, Lugo, Spain

**Keywords:** Neuroendocrine tumors, PRRT, [^177^Lu]Lu-DOTA-TATE, Delphi consensus, Somatostatin receptors, PET SSTR, Follow-up

## Abstract

**Background:**

Peptide receptor radionuclide therapy (PRRT) is an established treatment for patients with well-differentiated gastroenteropancreatic neuroendocrine tumors (GEP-NETs) expressing somatostatin receptors (SSTR). Despite robust trial and real-world data, heterogeneity persists regarding patient selection, therapeutic sequencing, and follow-up strategies.

**Methods:**

A Delphi consensus was conducted by the Galician Multidisciplinary Group on Neuroendocrine and Endocrine Tumors (GGNET). Ten experts in oncology, endocrinology, nuclear medicine, and radiology participated. 29 clinical statements were developed after a systematic review and rated using a 4-point Likert scale. Consensus was defined as ≥ 70% agreement.

**Results:**

Consensus (defined a priori as ≥ 70% agreement) was achieved for all statements although the level of agreement varied across domains. PRRT was endorsed for patients with unresectable or metastatic, progressive, well-differentiated GEP-NETs (grades 1–3, Ki-67 ≤ 55%) with confirmed SSTR expression. SSTR-targeted imaging (PET or scintigraphy) was considered mandatory for eligibility, with PET identified as the preferred modality. [^18^F]-FDG-PET was recommended selectively as a complementary prognostic tool in higher-grade tumors, rapid progression, or discordant imaging. Multidisciplinary tumor board review was universally supported. Guidance was provided on treatment administration, including standard dosing, renal protection, hematologic monitoring, and individualized risk assessment. Routine interim imaging was not recommended. Structured follow-up with CT/MRI was endorsed, with indication-driven use of SSTR or FDG-PET and limited routine value of non-specific biomarkers. Functional biomarkers, such as 5-HIAA and peptide hormones, retained utility in functioning tumors.

**Conclusions:**

This Delphi consensus provides pragmatic, multidisciplinary, and evidence-informed guidance to harmonize routine clinical practice in the use of PRRT for well-differentiated, SSTR-positive NETs. The proposed statements and the algorithm aim to harmonize practice across centers, reduce variability in care, enhance safety, and ultimately improve patient outcomes.

## Introduction

Neuroendocrine tumors (NETs) are a heterogeneous group of rare neoplasms derived from neuroendocrine cells, most commonly located in gastrointestinal tract, pancreas, bronchopulmonary system, thymus, and adrenal or extra-adrenal paraganglionic tissue (pheochromocytomas and paragangliomas) [1]. According to the 2022 World Health Organization (WHO) classification, NETs are defined as well-differentiated epithelial neoplasms stratified into three grades (G1–G3) based on mitotic count and Ki-67 index, with location-specific cut-offs. By contrast, neuroendocrine carcinomas (NECs) are poorly differentiated neoplasms classified as grade 3 and subdivided into small-cell and large-cell types [2].

From a functional perspective, NETs are classified as functioning or non-functioning depending on the presence of hormone-related symptoms. Functioning NETs can cause distinct clinical syndromes with a significant impact on quality of life, whereas non-functioning tumors are often diagnosed in advanced stages due to mass effect or metastatic disease [3].

Management of NETs is complex due to their biological heterogeneity, variable clinical behavior, and differences in grade, site, and disease extent [4]. Surgical resection remains the treatment of choice in localized stages, as it offers the potential for long-term disease control. However, many patients are diagnosed with unresectable or metastatic disease, requiring systemic therapy and individualized management [5]. Given the diversity of clinical scenarios, treatment decisions should ideally be guided by a multidisciplinary tumor board with expertise in neuroendocrine neoplasms.

Somatostatin analogs (SSAs) are the standard first-line treatment for well-differentiated NETs with somatostatin receptor (SSTR) expression, providing anti-proliferative effects and control of hormone-related symptoms in functioning tumors [6, 7]. The high prevalence of SSTR expression—reported in more than 80% of NETs—also enables molecular imaging and supports the use of peptide receptor radionuclide therapy (PRRT) as a targeted option [8–12].

PRRT with [^177^Lu]Lu-DOTA-TATE has emerged as a cornerstone treatment for patients with SSTR-positive NETs. The pivotal NETTER-1 trial demonstrated a significant improvement in progression-free survival (PFS) and objective response rate (ORR) in patients with progressive mid-gut NETs previously treated with somatostatin analogs [8]. More recently, the NETTER-2 trial extended these findings by showing the benefit of PRRT in the first-line setting for grade 2–3 gastroenteropancreatic NETs (GEP-NETs), with improvements in both PFS and ORR [9]. Additional randomized evidence is becoming available from the phase III COMPETE trial—comparing [^177^Lu]Lu-edotreotide with everolimus in G1–G2 GEP-NETs—and from the OCCLURANDOM trial—comparing [^177^Lu]Lu-DOTA-TATE with sunitinib in G1–G3 pancreatic NETs—further consolidating the therapeutic role of PRRT across different clinical scenarios [10, 11].

Beyond randomized trials, large real-world series have reinforced and expanded the evidence base for PRRT. The Erasmus MC experience not only confirmed durable disease control and long-term safety but also provided valuable data in bronchopulmonary and thymic NETs, clinical settings not addressed in randomized studies [12]. Similarly, the SEPTRALU registry captured a broad spectrum of NETs in routine practice—including GEP, pulmonary, and rarer entities, such as pheochromocytomas and paragangliomas—thereby extending the applicability of PRRT beyond the classical GEP-NET population [13]. More recently, a phase II study by Lin et al. prospectively demonstrated meaningful antitumor activity of [^177^Lu]Lu-DOTA-TATE in progressive metastatic pheochromocytomas and paragangliomas [14].

Appropriate patient selection is critical to maximize the benefit of PRRT. Confirmation of SSTR expression by functional imaging—most commonly [^68^Ga]Ga-DOTA PET/CT or scintigraphy—is mandatory before treatment. In selected scenarios, particularly in higher-grade tumors, aggressive clinical behavior, or discordance between morphological and functional imaging, [^18^F]-FDG-PET provides complementary prognostic information and may support risk stratification, without constituting an exclusion criterion for PRRT [15, 16].

Despite the expanding evidence base, clinical practice remains heterogeneous with respect to candidate selection, imaging workflows, therapeutic sequencing, response evaluation, and follow-up. Importantly, this heterogeneity largely reflects differences in real-world workflows and clinical decision-making rather than a lack of efficacy data supporting PRRT. To address these gaps, the Galician Multidisciplinary Group on Neuroendocrine and Endocrine Tumors (GGNET) convened a Delphi panel to develop pragmatic, multidisciplinary, practice-oriented recommendations for PRRT in well-differentiated, SSTR-positive NETs—covering patient selection, treatment delivery with [^177^Lu]Lu-DOTA-TATE and post-treatment follow-up. The resulting statements and the algorithms are intended to harmonize practice across centers and support decision-making in routine care.

This Delphi consensus was conceived as a real-world, practice-oriented document. Rather than redefining PRRT indications or addressing investigational strategies, its primary objective was to provide operational guidance along the standard PRRT pathway—in areas where international guidelines appropriately remain non-prescriptive and where variability in daily practice persists.

## Material and methods

The recommendations presented in this document are the result of a comprehensive literature review and a structured consensus process based on the Delphi methodology. This Delphi initiative was conceived as a pragmatic, real-world, practice-oriented process aimed at harmonizing routine clinical management of PRRT across centers within a regional multidisciplinary network, rather than redefining indications or addressing investigational strategies. The expert panel comprised 10 specialists with recognized expertise in the management of neuroendocrine tumors, representing endocrinology (*n* = 3), nuclear medicine (*n* = 3), medical oncology (*n* = 3), and radiology (*n* = 1). All panelists were members of the GGNET.

Experts were selected among GGNET members based on predefined, objective eligibility criteria reflecting real-world clinical expertise in the management of neuroendocrine tumors and PRRT. Eligibility criteria included active involvement in multidisciplinary tumor boards managing NET patients, regular participation in clinical decision-making related to PRRT, and direct experience in the follow-up of patients treated with PRRT. Multidisciplinary representation and hands-on clinical experience were prioritized in panel selection, in line with the practice-oriented scope of the consensus. Participation in PRRT-related clinical trials, contribution to PRRT-relevant publications or guidelines, cumulative experience in PRRT administration, and annual clinical exposure to PRRT-treated patients were considered supportive indicators of expertise when applicable, but were not mandatory inclusion criteria.

A systematic literature search was conducted in PubMed, covering publications up to January 31, 2024. The search terms included: “management of NETs,” “treatment guidelines for NETs,” “PRRT in NETs,” “somatostatin receptor expression in NETs,” “evaluation of treatment with PRRT,” and “follow-up of patients with NETs.” National and international clinical guidelines, original studies, and previously published consensus documents were also reviewed. The results of this review were used to identify key domains of routine clinical practice and areas of variability relevant to PRRT delivery, which informed the development of the Delphi statements.

Based on this review, a structured questionnaire was designed, including statements on patient selection, treatment administration, response assessment, imaging, biomarker use, and follow-up after PRRT. The statements were generated by a multidisciplinary core group comprising one medical oncologist, one nuclear medicine physician, and one endocrinologist, who drafted the initial items based on clinical relevance and identified practice gaps. In February 2024, the questionnaire was distributed to all panelists, who responded individually and anonymously using a structured Likert scale.

A single-round Delphi survey was conducted. Consensus was predefined as agreement by at least 70% of panelists rating a statement as “agree” or “strongly agree.” Following completion of the survey, a structured face-to-face consensus meeting was held on March 18, 2024, with a duration of approximately three hours. During this meeting, all statements were discussed in detail to contextualize their clinical applicability and to address areas of disagreement.

Statements that did not initially reach the predefined consensus threshold were re-voted after discussion. No statements were reformulated, merged, or eliminated during the process, as the discussion focused on interpretation and practical implementation rather than modification of item content. An external company provided logistical support for the face-to-face meeting but did not participate in item generation, voting, or interpretation of the results.

The final recommendations and the level of agreement for each statement are summarized in Table 1. Detailed agreement percentages are reported for each statement, allowing both consensus and variability across items to be explicitly assessed.

**Table 1:** List of recommendations and degree of agreement of the expert panel (1: strongly disagree; 2: disagree; 3: agree; 4: strongly agree)

Recommendations	Level of agreement			
Patient selection	1	2	3	4
Indications				
Statement 1. Any patient with a well-differentiated, grade 1–3 (Ki67 ≤ 55%), SSTR-positive, progressive, unresectable or metastatic GEP-NET is a potential candidate for peptide receptor-targeted radionuclide therapy (PRRT)	0	0	10%	90%
Statement 2. PRRT should be assessed on a case-by-case basis in:				
2.1: Bronchopulmonary or thymic NETs	0	10%	0	90%
2.2: Pheochromocytoma-paragangliomas	0	0	10%	90%
2.3: Other neoplasms with SSTR expression	0	0	10%	90%
Statement 3. The eligibility of a patient to receive PRRT should be discussed in a multidisciplinary committee	0	0	0	100%
Somatostatin receptor expression				
Statement 4. A patient is deemed a candidate for PRRT if lesions detected on morphological imaging tests have positive uptake on functional imaging with somatostatin analogues (PET-SSTR or scintigraphy)	0	10%	20%	70%
Statement 5. A lesion is considered to be SSTR positive if its uptake is higher than that of the liver in the case of PET-SSTR or [^111^In]-pentetreotide or [^99m^Tc]Tc-octreotide scintigraphy	0	0	0	100%
Statement 6. PET-SSTR is considered the functional imaging test of choice for determining SSTR expression	0	0	0	100%
Statement 7. If PET-SSTR is not accessible, the following alternatives are considered valid:				
7.1. Referral of the patient to a center with PET-SSTR availability *(preferred option was considered)*	0	0	0	100%
7.2. *If the above was not possible*: the performance of [^111^In]In-pentetreotide or [^99m^Tc]Tc-octreotide scintigraphy	0	0	30%	70%
Statement 8. PET-SSTR may be considered in patients with previous negative [^111^In]In-pentetreotide or [^99m^Tc]Tc-octreotide scintigraphy	0	0	10%	90%
Usefulness of [^18^F]-Fluorodeoxyglucose *([*^*18*^*F]-FDG)* PET				
Statement 9. The *[*^*18*^*F]-FDG* PET complements the information from the PET-SSTR, identifying tumor heterogeneity and optimizing the selection of patients who are candidates for PRRT	0	10%	0	90%
Statement 10. The performance of FDG-PET could be particularly useful in the following scenarios:				
10.1. Discordance between imaging tests for SSTR and morphological imaging tests	0	0	0	100%
10.2. Rapid clinical or radiological progression (< 6 months) to previous therapies administered	0	0	0	100%
10.3. Well-differentiated grade 3 or high grade 2 NETs (Ki 67 > 5%)	0	0	0	100%
Other parameters				
Statement 11. Patients who are candidates for PRRT must be in good overall health and have adequate bone marrow reserve, kidney, and liver function	0	0	30%	70%
Statement 12. The risk–benefit ratio of PRRT administration should be assessed in patients with the following conditions:				
12.1. Kidney failure (creatinine clearance less than 40 ml/min)	0	0	10%	90%
12.2. Low bone marrow reserve (leukocytes < 2 g × 10^9^/L, Hb < 8 g/dL or platelets < 75 × 10^9^/L)	0	0	10%	90%
12.3. Impaired liver function (bilirubin > 3 times normal, albumin < 3 g/dL or INR > 1.5)	0	10%	10%	80%
12.4. Extensive peritoneal carcinomatosis	0	0	10%	90%
Statement 13. Close monitoring for myelotoxicity is recommended in high-risk patients:				
13.1. Older than 70 years	0	0	30%	70%
13.2. Kidney failure (clearance 40–60 ml/min)	0	0	10%	90%
13.3. Presence of previous cytopenias	0	0	0	100%
13.4. Extensive metastatic bone disease (> 50% of the skeleton)	0	0	0	100%
13.5. Prior chemotherapy, especially with alkylating agents	0	0	0	100%
13.6. Previous radiotherapy	0	0	10%	90%
Treatment				
Statement 14. Laboratory tests (including at least CBC, kidney and liver function) should be performed 2 weeks before the first cycle, at 4 and 6 weeks after each cycle, and at 3, 6 and 12 months after completion of treatment	0	0	10%	90%
Statement 15. Routine mid-treatment morphological or functional imaging tests are not recommended. They should be considered in the following cases:				
15.1. Clinical deterioration	0	0	10%	90%
15.2. Impaired liver function	0	10%	10%	80%
15.3. Patients at high risk of progression (high grade, high tumor burden, rapid progression to previous lines…)	0	10%	0	90%
Response assessment and follow-up				
Morphological imaging tests				
Statement 16. Morphological imaging tests, including multiphasic thoracoabdomino-pelvic computed tomography (CT) and/or magnetic resonance imaging (MRI), represent the gold standard for response assessment and follow-up after PRRT	0	10%	10%	80%
Statement 17. Response evaluation shall be performed using the RECIST 1.1 response evaluation criteria	0	0	40%	60%
Statement 18. Morphological imaging tests will be performed at 3 and 6 months after the end of PRRT and every 6 months thereafter	0	0	10%	90%
Statement 19. The frequency of morphological imaging may be modified according to patient-related factors and/or tumor aggressiveness	0	0	0	100%
Functional imaging tests				
Statement 20. Functional imaging for somatostatin receptors should be performed 9–12 months after completion of PRRT	0	0	10%	90%
Statement 21. It is not recommended to perform routine periodic somatostatin receptor imaging at follow-up after PRRT	0	30%	10%	60%
Statement 22. Imaging for SSTR could be considered in follow-up after PRRT therapy in the following cases:				
22.1. Clinical deterioration	0	0	10%	90%
22.2. Suspected progression on morphological imaging	0	0	10%	90%
22.3. Small primary tumors	0	0	20%	80%
22.4. Nodal, bone or infiltrative disease	0	0	20%	80%
Statement 23. Changes in PET-SSTR lesion avidity (SUV) should not be considered as the sole criterion for decision making in response assessment or follow-up	0	0	10%	90%
Statement 24. Periodic [^18^F]-FDG PET scans are not routinely recommended for follow-up after PRRT	10%	0	10%	80%
Statement 25. [^18^F]-FDG PET could be useful:				
25.1. To resolve discrepancies between morphological and functional imaging findings for SSTR	0	0	10%	90%
25.2. When tumor dedifferentiation is suspected	0	0	0	100%
Statement 26. It is recommended that the same SSTR imaging modality should be used before and after PRRT			20%	80%
Circulating response biomarkers				
Statement 27. The routine use of non-specific circulating biomarkers, such as chromogranin A, in response assessment and follow-up after PRRT is not recommended	0	10%	40%	50%
Statement 28. The determination of 5-hydroxyindoleacetic acid (5-HIAA) in small bowel NETs with associated carcinoid syndrome may be useful	0	0	0	100%
Statement 29. Determination of specific neuropeptides could be useful in case of functioning pancreatic NETs	0	0	0	100%

*5-HIAA* 5-hydroxyindoleacetic acid, *FDG* ^18^F-fluorodeoxyglucose, *GEP* gastroenteropancreatic, *Hb* hemoglobin, *INR* international normalized ratio, *PET* positron emission tomography, *PRRT* peptide receptor radionuclide therapy, *SSTR* somatostatin receptor, *SUV* standardized uptake value, *CT* computed tomography, *NET* neuroendocrine tumor

## Results

All 10 invited experts completed the questionnaire and participated in the consensus meeting. Consensus was defined a priori as agreement by at least 70% of panelists. Overall agreement was high, and consensus (≥ 70% agreement) was achieved for all proposed statements. However, the degree of agreement was not uniform across items. Several statements reached consensus with lower proportions of “strongly agree” responses, indicating areas of residual clinical uncertainty rather than uncontested standards of care. The complete set of statements and their corresponding levels of agreement are summarized in Table 1, which reports detailed agreement percentages for each item and provides a structured framework for the clinical use of PRRT in neuroendocrine tumors.

In the patient selection domain, the panel agreed on the need to confirm somatostatin receptor expression by functional imaging, recognized the complementary prognostic (rather than exclusionary) role of [^18^F]-FDG-PET/CT in selected cases, and highlighted the influence of clinical and pathological features, such as tumor grade and primary site.

For treatment administration, the experts endorsed standard dosing, the use of renal protection, and close monitoring for hematological toxicity, particularly in high-risk patients. The role of multidisciplinary tumor boards was also emphasized as a key element in individualizing therapeutic decisions.

In the response assessment domain, agreement was reached on the use of an integrated approach combining morphological criteria (RECIST), functional imaging, and biochemical markers when appropriate. The level of agreement across statements in this domain varied, underscoring the complexity of response evaluation after PRRT.

Finally, in the follow-up domain, the panel supported consensus on the type and periodicity of imaging after PRRT, together with long-term surveillance strategies to detect late toxicities and monitor disease progression. As reflected in Table 1, agreement levels also differed across follow-up-related statements, highlighting areas where clinical practice remains heterogeneous despite shared principles.

In the response assessment domain, agreement was reached on the use of an integrated approach combining morphological criteria (RECIST), functional imaging, and biochemical markers when appropriate. The level of agreement across statements in this domain varied, underscoring the complexity of response evaluation after PRRT and the absence of a single standardized approach in routine practice.

Finally, in the follow-up domain, the panel supported consensus on the type and periodicity of imaging after PRRT, together with long-term surveillance strategies to detect late toxicities and monitor disease progression. As reflected in Table 1, agreement levels also differed across follow-up-related statements, highlighting areas where clinical practice remains heterogeneous despite shared principles.

## Discussion

This Delphi consensus provides a comprehensive framework for the clinical use of PRRT in well-differentiated NETs, integrating evidence from randomized trials, real-world studies, and expert opinion. Importantly, this consensus should be interpreted in light of its intended scope. By organizing the recommendations into key domains—patient selection, imaging strategies, treatment administration, response assessment, and follow-up—this work seeks to harmonize practice in an area of considerable clinical heterogeneity. The Delphi process was conceived as a pragmatic, real-world, practice-oriented initiative aimed at harmonizing day-to-day clinical management of PRRT across centers, rather than redefining formal indications or addressing non-standard or investigational strategies. The consensus builds on pivotal randomized studies, recent phase II and III trials, and registry-based cohorts, providing guidance for established indications as well as for emerging scenarios, such as pheochromocytomas and paragangliomas.

Rather than establishing new formal indications, these recommendations should be regarded as supportive guidance in selected situations, particularly in bronchopulmonary NETs, pheochromocytomas/paragangliomas, and well-differentiated grade 3 tumors (Ki-67 ≤ 55%). This positioning reflects routine clinical practice in specialized centers rather than an expansion of approved indications. In the latter group, although the NETTER-2 trial demonstrated significant benefit of [^177^Lu]Lu-DOTA-TATE in untreated G2–3 GEP-NETs with Ki-67 up to 55% [9], regulatory approval by the EMA and FDA is still pending, and treatment should only be considered after individualized multidisciplinary discussion.

Accurate patient selection is fundamental to maximize the benefit–risk ratio of PRRT. Patients with well-differentiated GEP-NETs (grades 1–3, Ki-67 ≤ 55%), unresectable or metastatic, and with confirmed SSTR expression are considered potential candidates. The NETTER-1 trial demonstrated a median PFS of 28.4 months with [^177^Lu]Lu-DOTA-TATE compared with 8.4 months with high-dose octreotide, with an ORR of 18% vs 3% and a clinically relevant OS advantage despite crossover [8, 17]. More recently, the NETTER-2 trial showed that in previously untreated G2–3 GEP-NETs (Ki-67 10–55%), first-line PRRT achieved a median PFS of 22.8 vs 8.5 months and an ORR of 43% vs 9% [9]. In the COMPETE phase III trial, [^177^Lu]Lu-edotreotide demonstrated superiority over everolimus in G1–2 GEP-NETs, with a median PFS of 21.3 vs 9.4 months and higher disease control rates [10]. In pancreatic NETs, the OCLURANDOM trial reported a median PFS of 20.7 vs 11.0 months with PRRT compared with sunitinib, with an ORR of 28% vs 10% and a more favorable safety profile [11]. Real-world cohorts, including the Erasmus MC experience, confirmed durable disease control, with a median PFS of 29 months, OS exceeding 60 months, and long-term safety characterized mainly by manageable hematologic and renal toxicity [12].

Beyond digestive primary NETs, PRRT has shown clinically relevant activity in other NET subtypes. In the Erasmus MC series, patients with bronchopulmonary NETs achieved a median PFS of 21.6 months and OS of 52.3 months, confirming durable disease control beyond the GEP setting [12]. Similarly, the SEPTRALU registry broadened real-world evidence across multiple NET subtypes: in this cohort, bronchopulmonary tumors reached a median PFS of 17.6 months, while patients with pheochromocytomas and paragangliomas achieved a median PFS of 30.6 months and partial responses in more than 30% of cases, with favorable tolerability [13]. Prospective confirmation was provided by the phase II trial of Lin et al., where [^177^Lu]Lu-DOTA-TATE induced disease control in over 80% of progressive metastatic pheochromocytomas and paragangliomas [14]. These data support the cautious use of PRRT in selected non-GEP-NETs within a multidisciplinary framework, despite the absence of randomized phase III evidence in these populations. In parallel, the ongoing LEVEL trial is specifically evaluating PRRT in bronchopulmonary NETs, which will be instrumental to consolidate its role in these rare but clinically relevant entities.

Eligibility for PRRT should always be reviewed in a multidisciplinary tumor board. This approach, endorsed by ENETS and NCCN guidelines [5, 18], ensures adequate integration of imaging, optimal sequencing with other systemic therapies, and thorough evaluation of comorbidities and patient preferences, ultimately aiming to maximize therapeutic benefit.

Confirmation of somatostatin receptor expression is an essential prerequisite for PRRT, as its efficacy relies on radionuclide uptake and internalization by tumor cells. In NETTER-1, eligibility required a Krenning score ≥ 2 on [^111^In]-DTPA-octreotide scintigraphy, equivalent to uptake at least comparable to the liver [8], a criterion subsequently endorsed by international guidelines [19, 20]. NETTER-2 confirmed that robust SSTR expression remains mandatory in earlier treatment lines for G2–3 GEP-NETs (Ki-67 10–55%), using either scintigraphy or SSTR-PET to document uptake above liver background [9].

Over the past decade, PET imaging with radiolabeled somatostatin analogs has largely replaced scintigraphy as the preferred tool for assessing receptor expression. SSTR-PET offers higher sensitivity and specificity, superior spatial resolution, and semi-quantitative parameters such as SUVmax or tumor-to-liver ratio, which correlate with treatment outcomes [21–23]. Beyond diagnostic accuracy, it has demonstrated a direct impact on management, with therapeutic strategies modified in up to 40% of patients when used after inconclusive conventional imaging [24]. On this basis, the consensus identified SSTR-PET as the standard for confirming eligibility, recommending referral to specialized centers whenever local access is limited. In parallel,

In parallel, [^18^F]-FDG-PET/CT provides complementary prognostic information in selected scenarios. The panel deliberately positioned FDG-PET as a complementary prognostic tool rather than as an exclusion criterion for PRRT eligibility, in line with the design of pivotal PRRT trials. Dual-tracer imaging enables biological phenotyping, with high FDG uptake identifying tumors with more aggressive behavior and poorer outcomes [15, 25]. This is particularly relevant in higher-grade well-differentiated NETs, rapid clinical progression, or discordant findings between morphology and receptor imaging. Its use, however, should not be universal but restricted to cases with clinical or pathological features suggestive of unfavorable biology. Given the absence of validated thresholds defining FDG dominance and the lack of prospective evidence supporting FDG-driven treatment algorithms, the consensus deliberately avoided proposing rigid operational cut-offs. The systemic management of well-differentiated GEP-NETs with SSTR expression usually begins with long-acting SSAs as standard first-line therapy [6, 7]. In patients with disease progression, PRRT with [^177^Lu]Lu-DOTA-TATE is an established option supported by randomized and real-world evidence; however, differences in schedules and concomitant SSA use across trials warrant caution when extrapolating to routine care [8–13]. In daily practice, [^177^Lu]Lu-DOTA-TATE is generally administered as four cycles every 8 weeks at a fixed activity of 7.4 GBq, with continuation of background SSA for hormonal control and as part of the therapeutic scheme. This NETTER-1–aligned regimen has been consistently reproduced in real-world cohorts, supporting feasibility and safety [8, 12, 13].

In this context, close monitoring is essential to ensure safety and guide adjustments. Laboratory testing (complete blood count, renal function, and liver profile) is recommended 2 weeks prior to the first cycle, before each subsequent cycle, and again 4–6 weeks after administration. After completing therapy, follow-up assessments at 3, 6, and 12 months and then annually are advised, with interval individualization according to tolerance, comorbidities, or unexpected abnormalities [26, 27]. Particular vigilance for hematological toxicity is warranted in high-risk patients, such as those older than 70 years, with baseline cytopenia, prior chemotherapy or radiotherapy, extensive bone metastases, or moderate renal impairment. Importantly, no comparative evidence supports prioritization of dose reduction, interval extension, or temporary treatment interruption over another; therefore, management strategies should be individualized rather than algorithmic. A careful risk–benefit assessment is mandatory in cases with severe renal dysfunction, impaired marrow reserve, or relevant hepatic dysfunction [26, 27].

Routine interim imaging during PRRT is not recommended as it rarely changes management [28]. Available data do not demonstrate a clear clinical benefit of systematic interim imaging, and early assessments may be confounded by delayed responses or pseudoprogression. It may be considered in cases of clinical deterioration, impaired liver function, or high-risk disease features (e.g., higher grade, large tumor burden, or rapid progression to prior therapies), balancing the need for decision-making against the risk of misinterpretation [29, 30].

After completion of PRRT, a structured follow-up is essential to evaluate treatment response, detect disease progression, and monitor late toxicities. Morphological imaging remains the cornerstone of surveillance. Multiphasic contrast-enhanced CT or MRI allows accurate assessment of tumor size and vascularity, particularly relevant in slowly growing neoplasms such as NETs [31]. Response is usually evaluated according to RECIST 1.1, which, despite limitations in indolent tumors and atypical patterns such as pseudoprogression, remains the most reproducible tool in clinical practice and trials [32, 33]. The proposed imaging schedule reflects a pragmatic approach aligned with routine NET surveillance rather than a PRRT-specific evidence-based optimal timing. Most guidelines and expert recommendations suggest imaging at 3 and 6 months after the last cycle of PRRT, followed by surveillance every 6 months, with interval adjustment according to clinical features, tumor aggressiveness, or patient-related factors [31].

Functional imaging with SSTR-targeted techniques, either PET or scintigraphy, provides complementary information. Although not recommended for routine follow-up, performing SSTR imaging 9–12 months post-therapy can establish a new baseline [28]. Thereafter, its use should be indication-driven rather than protocolized [24]. Changes in uptake intensity (SUVmax or visual grading) should not be used as stand-alone criteria, given variability in receptor expression and confounders such as concomitant SSA therapy [28]. In patients with aggressive disease biology or discordant findings, [^18^F]-FDG-PET can identify dedifferentiated clones with limited SSTR expression and provide prognostic information [15, 25]. Whenever possible, the same SSTR-based imaging modality should be used before and after PRRT to ensure consistency [30].

The role of circulating biomarkers is more limited. Chromogranin Ahas low sensitivity and specificity and is not recommended for routine PRRT response assessment [26]. In functioning tumors, hormone-specific markers remain clinically useful. In functioning mid-gut NETs, measurement of 5-HIAA in plasma or 24-h urine remains valuable for monitoring serotonin secretion, with decreases after therapy correlating with improved symptom control and reduced risk of carcinoid heart disease [26]. In functioning pancreatic tumors, determination of peptide hormones such as insulin, gastrin, glucagon, somatostatin, or VIP can also provide clinically meaningful information for monitoring and early relapse detection [3]. Notably, agreement among panelists was lower for statements related to follow-up imaging and non-specific biomarkers, reflecting ongoing heterogeneity in real-world practice and limitations of the available evidence.

The strengths of this consensus include its multidisciplinary composition, alignment with both randomized trials and real-world evidence, and the achievement of high agreement across domains. Limitations relate to reliance on expert opinion where data remain scarce, variability in access to specialized imaging and nuclear medicine resources, and the evolving nature of ongoing trials that may further refine indications and management strategies. Despite these challenges, the recommendations provide actionable, harmonized, and evidence-informed guidance to support the safe and effective use of PRRT in patients with well-differentiated, SSTR-positive NETs.

In summary, this Delphi consensus reflects deliberate, practice-oriented choices in areas where high-level evidence is lacking and where international guidelines appropriately remain non-prescriptive. Rather than issuing rigid or investigational recommendations, the panel sought to distinguish established standards from unresolved clinical questions and provide pragmatic guidance to support multidisciplinary decision-making in routine PRRT practice.

## Conclusions

This Delphi consensus by the GGNET provides pragmatic, multidisciplinary, expert-based recommendations for the selection, administration, and follow-up of patients undergoing PRRT. The panel emphasizes the central role of SSTR-PET imaging in establishing eligibility, the complementary (non-exclusionary) value of [^18^F]-FDG-PET in selected scenarios, and the importance of multidisciplinary evaluation to optimize the sequencing of systemic therapies and overall patient outcomes.

**Fig. 1:**
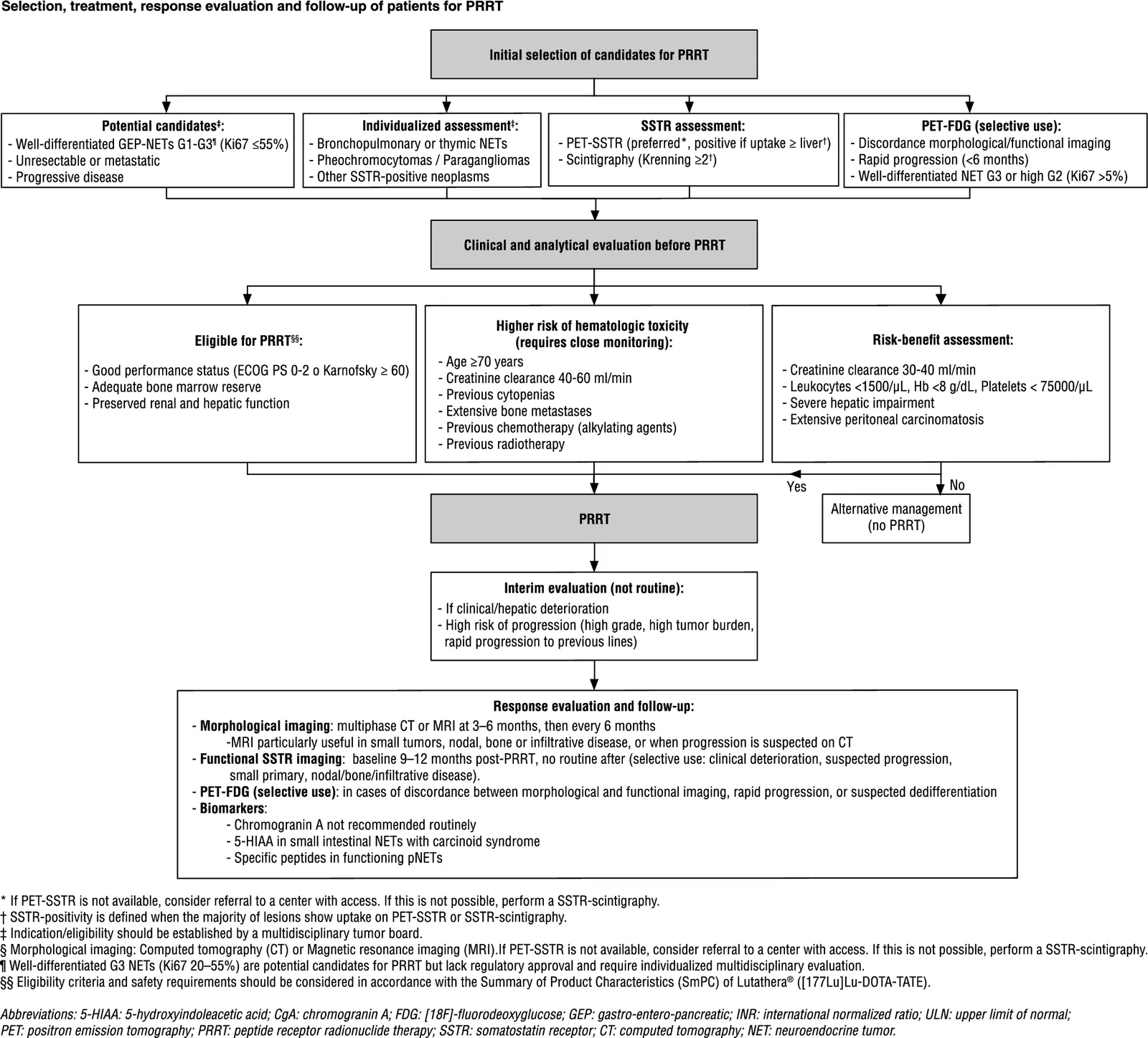
Selection, treatment, response evaluation and follow-up of patients for PRRT

The proposed algorithm (Fig. 1) offers a structured and pragmatic framework to harmonize practice across centers, supporting routine clinical decision-making by integrating evidence from randomized trials, real-world cohorts, and expert opinion. Rather than redefining indications, this work aims to reduce variability in care and support decision-making in day-to-day clinical practice.

Despite robust evidence supporting PRRT in GEP-NETs, uncertainties persist regarding its role in non-GEP primaries, the utility of circulating and functional biomarkers, and the optimal operational use of PRRT within standard treatment pathways, including patient selection and follow-up strategies. Continued prospective research is warranted to refine patient selection, optimize treatment schedules, and better define the long-term impact of PRRT.

## Data Availability

Not applicable.
